# Fast free-of-acrylamide clearing tissue (FACT)—an optimized new protocol for rapid, high-resolution imaging of three-dimensional brain tissue

**DOI:** 10.1038/s41598-017-10204-5

**Published:** 2017-08-29

**Authors:** Na Xu, Amin Tamadon, Yaan Liu, Tong Ma, Rehana K. Leak, Jun Chen, Yanqin Gao, Yi Feng

**Affiliations:** 1 State Key Laboratory of Medical Neurobiology and Institute of Brain Science, Brain Science Collaborative Innovation Center, Fudan University, Shanghai, 200032 China; 2 Department of Integrative Medicine and Neurobiology, School of Basic Medical Sciences, Shanghai Medical College, Institutes of Integrative Medicine of Fudan University, Shanghai, 200032 China; 3 Pittsburgh Institute of Brain Disorders and Recovery and Department of Neurology, University of Pittsburgh, Pittsburgh, PA 15213 USA; 4 Division of Pharmaceutical Sciences, Mylan School of Pharmacy, Duquesne University, Pittsburgh, PA 15282 USA

## Abstract

Fast Free-of-Acrylamide Clearing Tissue (FACT) is a new sodium dodecyl sulfate (SDS)-based clearing protocol for the chemical clearing and imaging of brain tissue containing transgenic or immunolabeled fluorescent proteins. In the present study, we have developed this new method and optimized multiple dimensions of the workflow, including reduced clearing time, improved efficiency of fluorescent signals without the need for electrophoretic or complex instrumentations, preservation of cytoarchitectural details, optimized confocal microscopy, and accelerated data collection. We systematically compared seven clearing protocols with the FACT protocol, using transgenic mouse brains with fluorochrome expression in microglia. Only six days were required for detecting transgene-labeled markers in a 1-mm thick brain slice from adult mice, and 14 days were required for detecting antibody-labeled markers in the same-sized tissue. Preservation of fluorescent signal was achieved by decreasing clearing time, adjusting the pH of the SDS solution, and using the appropriate temperature for tissue clearing, all of which contributed to the superiority of our method. We conclude that the FACT protocol can be successfully applied to the fluorescent imaging of mouse brain tissue, and will facilitate structural analyses and connectomics of large assemblies of cells and their networks in the context of three-dimensional organ systems.

## Introduction

Microglia are the resident parenchymal myeloid cells of the central nervous system (CNS). They play important roles in the development of the CNS, including the refinement and sculpting of synaptic networks during development^1–3^ and immune surveillance and defense against neurodegenerative diseases and neural injuries^4^. Therefore, understanding the complex interactions between microglia and other cell types is essential for defining their roles in the CNS. Because most neurodegenerative diseases affect a multitude of brain regions, the traditional method of tissue evaluation using two-dimensional imaging does not provide a comprehensive picture of cellular reactions to injury and intercellular interactions between neighboring or distant cells in three dimensions. Therefore, new and improved methods are urgently needed for the simultaneous evaluation of large populations of cells such as microglia in three dimensions, with a focus on fine details of their cytoarchitecture and their structural contacts with surrounding cells^5^.

Because of their higher expression levels, transgenic fluorescent proteins have stronger and more visible signals than antibody-stained markers and require shorter tissue preparation times. Several methods have been developed for the large-scale imaging of transparent and intact tissues with an emphasis on brain neural circuits, including BABB^6^, Scale^7^, 3DISCO^8^, ClearT^9^, SeeDB^10^, CLARITY^11^, passive CLARITY^12^, PACT^13^, CUBIC^14, 15^, and FASTClear^16^.

Of these approaches, the ones that clear tissue by replacing the water in the tissue with organic solvents, such as BABB and 3DISCO, cannot prevent the quenching of fluorescent protein signals for longer than two days^6, 8, 10^. Therefore, these approaches are limited in their usefulness for long-term tissue preservation or prolonged imaging applications. To overcome this serious limitation, aqueous-based clearing approaches such as Scale, SeeDB, and ClearT have been developed, and these can prevent fluorescent quenching for approximately one week without any changes in tissue size^7, 9, 10^. However, these powerful approaches are restricted to transgenic labels in animal models.

To address these issues, hydrogel-based clearing methods, including CLARITY and PACT, have been introduced^13, 17^. These approaches provide conditions for antibody labeling of tissue markers, and they can also be used with transgenic labels in animal models. However, CLARITY uses electrophoretic tissue clearing (ETC) to extract lipids from large samples, and this results in the destruction of fine cellular structures^11^. The PACT^13^ and passive CLARITY^12^ methods have faster clearing speed and preserve the tissue structure by avoiding the use of ETC. However, for long-term imaging, the deformation of tissues caused by hydrogel expansion during clearing limits the usefulness of these powerful methods for evaluating fine structures such as microglia branches and neuronal processes.

As a further improvement, the FASTClear^16^ method avoids the use of hydrogel and is performed at 50 °C to increase the clearing speed compared to PACT. However, the FASTClear approach has been limited to antibody labeling^16^. Thus, it was necessary to develop an optimized method to clear thick fluorescent tissue by reducing the clearing time while optimizing the reagents and temperature so as to preserve the fluorochrome signal. In an attempt to preserve the structure of microglial cells in order to image their branches and sub-branches and to visualize their connections with neighboring cells, we developed a new method by merging and modifying the PACT and FASTClear approaches^13, 16^. Removing the hydrogel perfusion and embedding steps from the PACT method improved the speed of clearing, and decreasing the temperature in the FASTClear method to 37 °C and optimizing the clearing solution pH to 7.5 decreased the quenching of fluorescent transgenic labels. Thus, the present study is the first to describe a simple and rapid approach, Fast Free-of-Acrylamide Clearing Tissue (FACT), which provides optimal conditions for visualizing transgenic fluorescent proteins and antibody labeling of tissue markers (Figure S1). We have systematically compared FACT with the passive CLARITY, PACT, and FASTClear methods for the evaluation of microglia in the cerebral cortex of transgene-labeled or immunolabeled mouse brains. The FACT protocol is original and distinct from other protocols in that it improves the signal to noise ratio, depth of tissue penetration, speed of processing, long-term retention of fluorescent signal, and preservation of cytoarchitecture.

## Results

### Accelerated clearing time with FACT relative to seven other established methods

**Figure 1:**
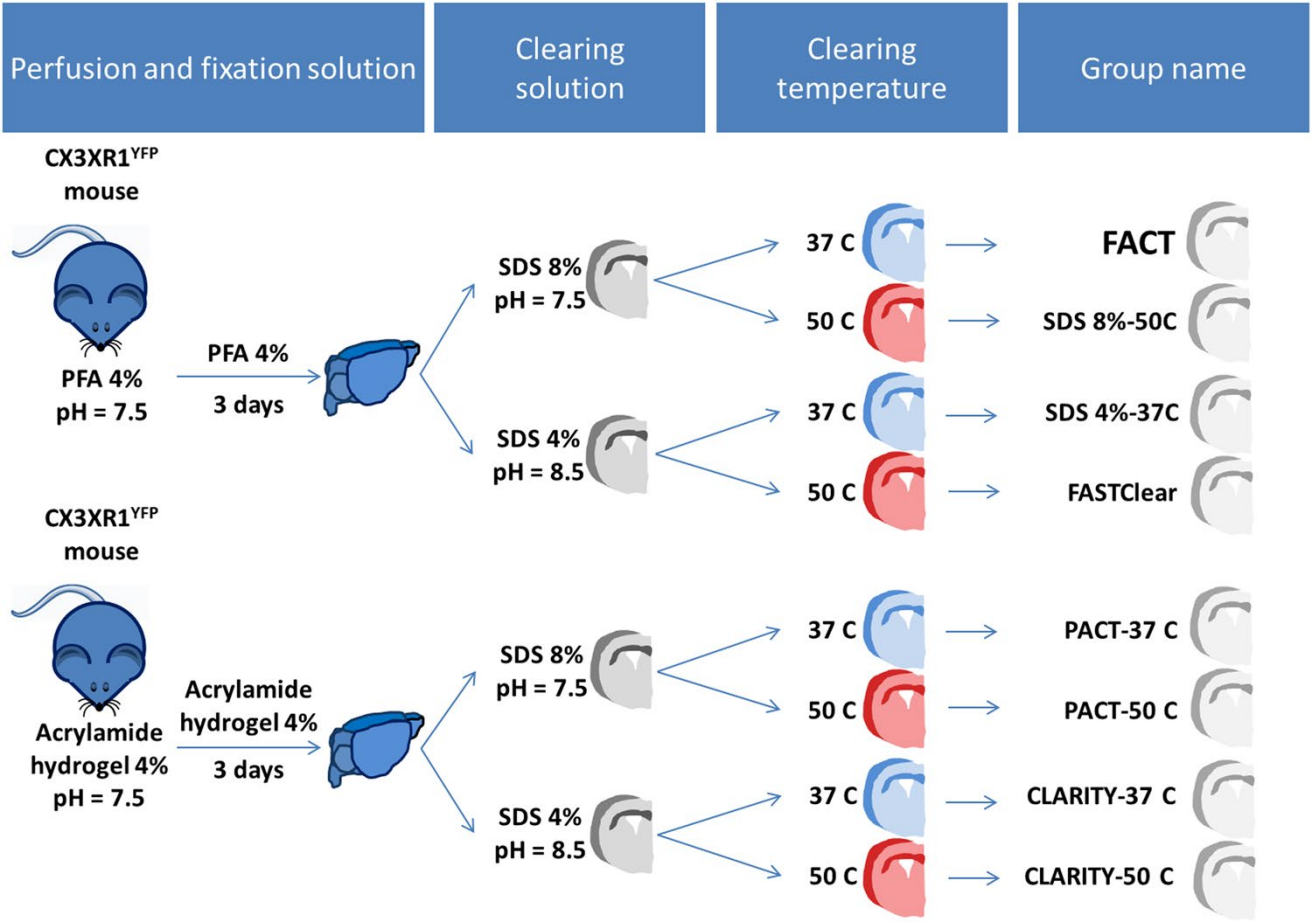
Pipeline overview of the Fast Free-of-Acrylamide Clearing Tissue (FACT) protocol in comparison with seven other protocols for clearing of mouse brain slices. After perfusion and fixation of brains with paraformaldehyde (PFA) or hydrogel solution, brain slices were allocated into four different clearing solutions with varying sodium dodecyl sulfate (SDS) concentrations and pH. The four subgroups were cleared at two different temperatures.

**Figure 2:**
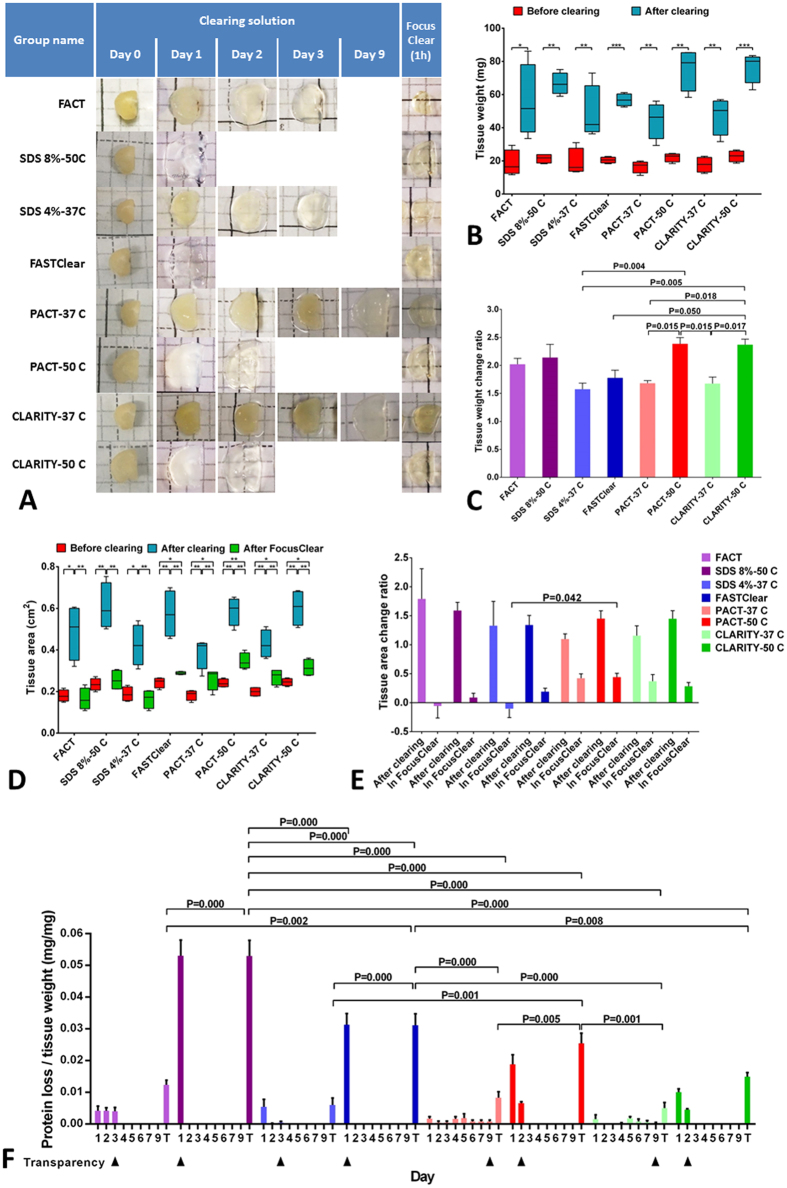
Comparison of clearing time, tissue size changes, and protein loss with eight protocols. (**A**) Optical transparency comparison of half of the 1 mm adult mouse sagittal blocks from eight different protocols cleared for different durations and after refractive index matching in FocusClear. (**B**) Box-whisker plots of tissue weight before and after clearing in each protocol, showing the smallest change with the FACT protocol (*p < 0.05; **p < 0.01; ***p < 0.001). (**C**) The means and standard errors of the weight change ratios in the brain sections for the eight groups are shown. There was an increase in weight in the hydrogel-based protocols and at higher temperatures. (**D**) Box-whisker plots of tissue area changes before and after clearing with each protocol and after refractive index matching with FocusClear. The smallest changes were seen for the FACT protocol and the SDS4%-37 °C protocol (*p < 0.05; **p < 0.01; ***p < 0.001). (**E**) Comparisons of means and standard errors of the tissue area change ratio across the eight groups. (**F**) Comparisons of daily and total (T) tissue protein loss measurements in the eight protocols. There was a reduction in protein loss at low temperatures. The arrowheads point to the day that transparency was achieved with each protocol.

In an attempt to find a quicker and simpler method for passive tissue clearing without destroying subtle cellular architecture, losing proteins, or losing fluorescent signal from transgenic proteins or antibodies, we systematically compared the FACT protocol with seven other approaches (Fig. 1). The brain slices were divided into 8 groups, and four were fixed with paraformaldehyde (PFA) to increase the clearing speed. The clearing speed of brain slices increases by increasing the temperature to 50 °C, and as shown in Fig. 2A, the brain slices were cleared in the SDS8%-50 °C and FASTClear groups within the first day and in the PACT-50 °C and CLARITY-50 °C groups after 2 days. In the 37 °C subgroups, removing hydrogel from the tissue caused slices in the FACT and SDS4%-37 °C group to clear within 3 days, while the hydrogel-embedded slices in the PACT-37 °C and CLARITY-37 °C groups were transparent only after 9 days. As depicted in Figure S1, the presence of polyacrylamide hydrogel in the intercellular and intracellular spaces was an obstacle for fast washing of lipids by SDS. In contrast, the absence of polyacrylamide hydrogel from the tissue fixation process in FACT and SDS4%-37 °C protocols facilitated lipid removal.

### Comparison of changes in tissue size with FACT versus seven other clearing methods

To compare the effect of FACT on tissue architecture with the other evaluated protocols, we measured tissue area (as an index of tissue size change) and tissue weight (as an index of water absorption and tissue expansion) after clearing. Comparison of tissue weights before and after clearing in each group (Fig. 2B) and the weight change ratio between groups (Fig. 2C) revealed that the most significant changes were in the hydrogel-embedded high-temperature groups. Importantly, the FACT weight change ratio was not significantly different from the other approaches such as FASTClear, PACT, or passive CLARITY.

Comparisons of tissue area before and after clearing and after FocusClear immersion (Fig. 2D) and comparisons of the area change ratio after clearing and after FocusClear immersion for 1 h (Fig. 2E) showed that FACT did not significantly change tissue area compared to FASTClear, PACT, and passive CLARITY, and the sizes of the tissues after shrinkage in FocusClear in the FACT, SDS4%-37 °C, and SDS8%-50 °C protocols were not significantly different compared to their sizes before clearing.

Increasing temperature or hydrogel concentration resulted in tissue expansion. Furthermore, the extracellular matrix and cytoskeleton can absorb water during the clearing process in the PFA-based protocols. Although expansion of the hydrogel can increase tissue volume and increase pore size to facilitate lipid removal (*e.g*. CLARITY and PACT), there is disruption of cellular architecture. Furthermore, the swelling is not reversible, even after using FocusClear, and adversely impacts tissue quality.

### Comparison of total protein loss with FACT, PACT-37 °C, and CLARITY-37 °C

Upon observing a significant increase in tissue transparency at higher temperatures and in non-hydrogel cleared brain sections, we assessed the total protein loss of all groups on a daily basis (Fig. 2F). Increasing the temperature to 50 °C and increasing the SDS to 8% increased protein loss in the brain slices. Interestingly, although removing hydrogel from the brain fixation procedure increased the daily protein loss in the FACT protocol compared to the CLARITY-37 °C and PACT-37 °C protocols, at the time of tissue transparency (day 3 for FACT and day 9 for CLARITY-37 °C and PACT-37 °C) the total protein losses were not significantly different between these groups.

As illustrated in Figure S1, PFA is expected to form strong bonds between proteins. The aldehyde reacts with one or two nitrogen or other atoms within peptides and proteins, and can create a -CH2- cross link known as a methylene bridge^18^. These and other connections illustrated in Figure S1 trap the proteins in place, including the transgenic proteins, and connect soluble cytosolic proteins with the proteins of the cytoskeleton. These bonds create a large three dimensional network that lends structural rigidity and tensile strength to the tissue during the harsh processing conditions. Some degree of protein loss is inevitable with all protocols, because some proteins may not be trapped within the intracellular space or extracellular matrix, such as extracellular or cytoplasmic membrane proteins. The increased speed of tissue clearing in FACT did not influence total protein loss, even in comparison with hydrogel-based approaches with the lowest clearing speed.

### Maximal preservation of fluorophore signal strength with FACT

**Figure 3:**
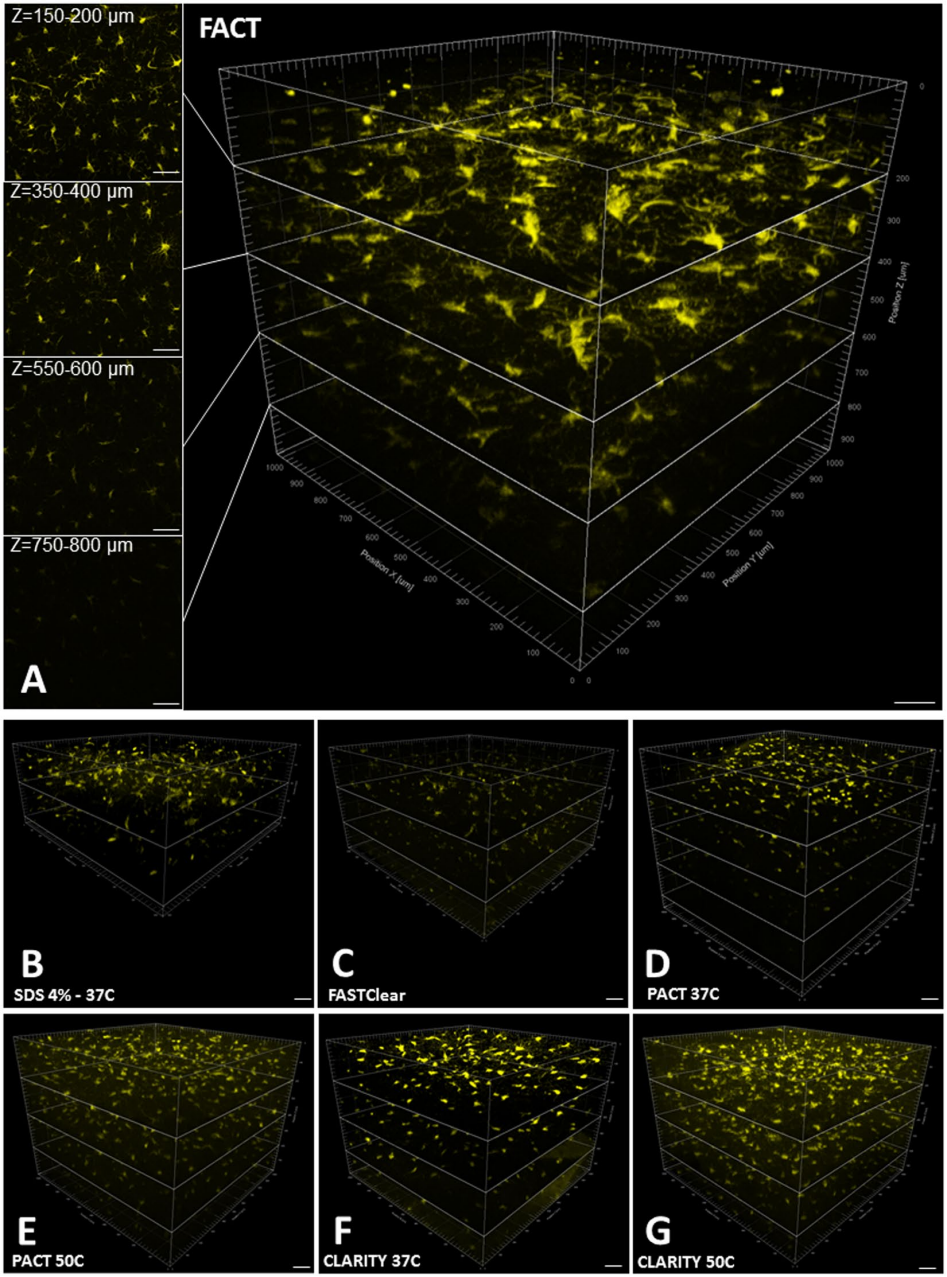
High-resolution imaging of microglia expressing YFP in the cerebral cortex after clearance with seven protocols. Confocal microscopy was employed at 25× magnification with an area of 1024 × 1024 μm^2^ and at different depths. (**A**) The XYZ acquisition of FACT-cleared cortex detected microglia to a depth of 948 μm (each section in Z is 200 μm). The left panels represent the maximum-intensity projections over a 50-µm-thick volume at different depths. (**B–G**) The other clearing protocols with different depths of microglia signal detection: SDS4%-37 °C, 442 μm; FASTClear, 561 μm; PACT-37 °C, 884 μm; PACT-50 °C, 783 μm; CLARITY-37 °C, 753 μm; and CLARITY-50 °C, 753 μm. Scale bars are 100 μm.

The most important issues for 3D tissue imaging include maximizing the fluorescent signal and the depth of the tissue that can be imaged. To compare the ability of the methods to preserve fluorescence signal, we scanned the 1-mm-thick brain cortex slices from CX3CR1^YFP^ mice using a confocal microscope under conditions of maximum signal and minimum background. The greatest depths at which the methods could detect fluorescence signal were observed with the FACT protocol, with a depth of Z = 800 μm (Fig. 3A and Video S1). The SDS 8%-50 °C group did not show any signal and was excluded from further evaluations. The signal in the SDS 4%-37 °C group was captured until Z = 400 μm, and the other methods all had maximum depths for optimized signal observation of Z = ~600 μm (Fig. 3B–G).

**Figure 4:**
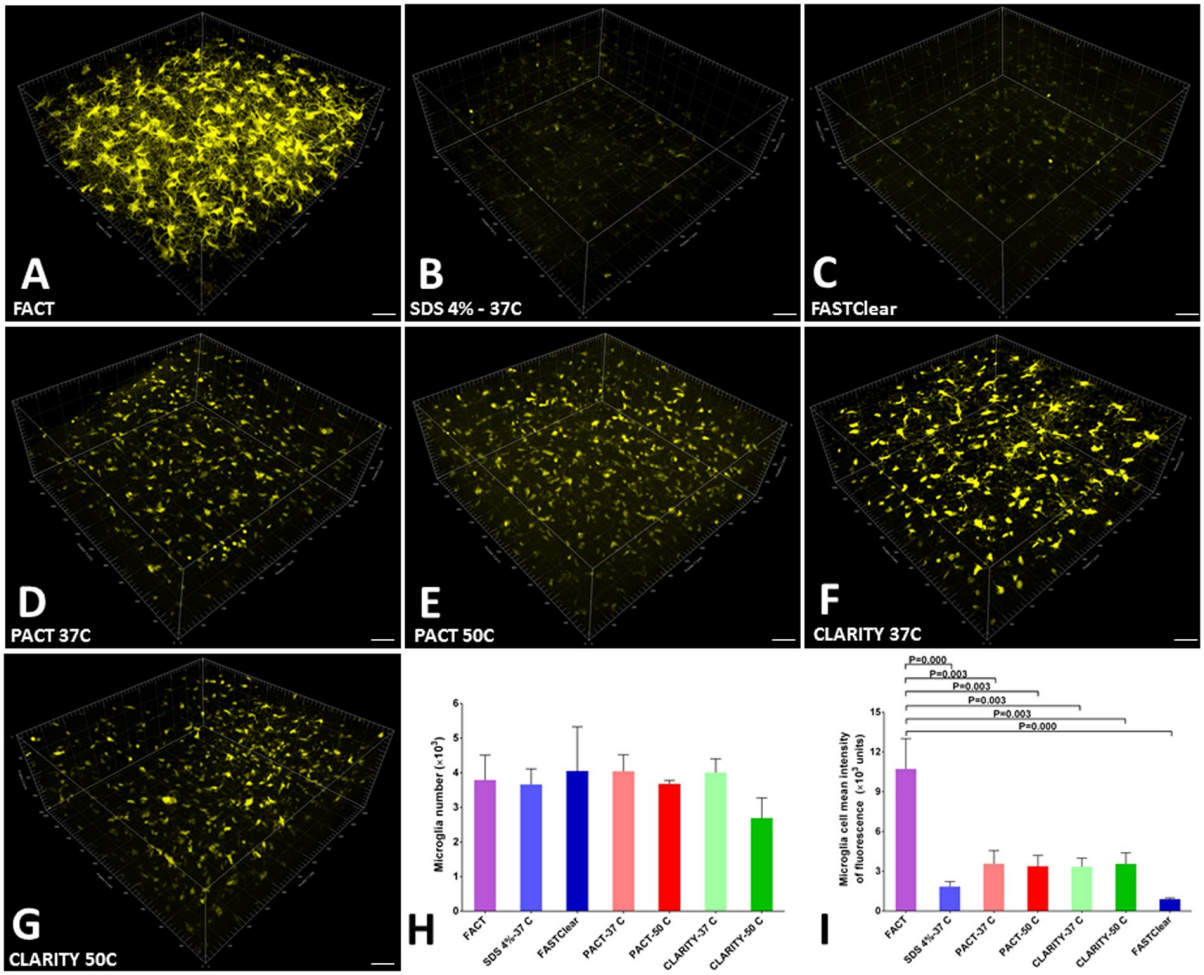
Comparisons of 400-µm-thick volumes of Z-stack images of microglia after testing seven protocols. (**A–G**) Three-dimensional blocks of microglia populations of the cerebral cortex in transgenic mice after clearing with the various protocols. The greatest signal and maximal architectural details were achieved with the FACT protocol. For all images, confocal microscopy was employed at 25**× **magnification. (**H**) Comparisons of corrected microglia numbers between the different clearing protocols (after semiautomatic counting with the Imaris Surface algorithm) reveal a lack of significant differences between the groups (*p* > 0.05). (**I**) Comparisons of the corrected mean intensities of the signals between the different protocols. The highest signal detection was achieved with the FACT protocol. Scale bars are 100 μm.

**Figure 5:**
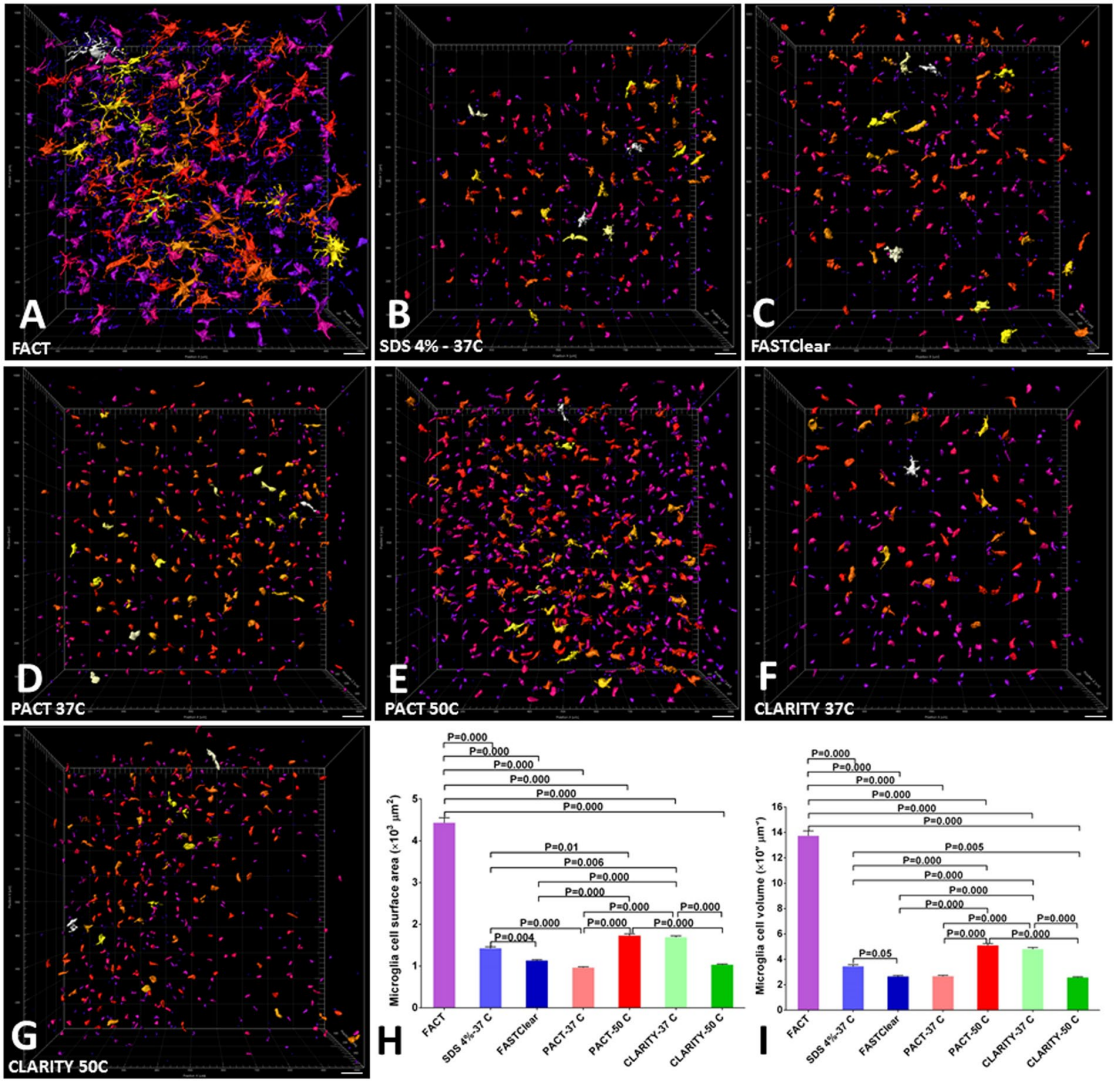
Three dimensional reconstructions of microglia in 400-µm-thick volumes of cerebral cortex after the different clearing protocols. (**A**–**G**) Different colors represent different surface areas of microglia reconstructed by the Imaris Surface algorithm and sorted by Imaris Vantage from white (highest area) to purple (lowest area). The maximum number of visible microglial structures was achieved with the FACT protocol. (**H** and **I**) Comparisons of microglial surface area and volume between the various protocols after semiautomatic measurement with the Imaris Surface algorithm. Maximum retention of YFP signal was achieved with the FACT protocol. Scale bars are 100 μm.

In addition to the maximum depth as an index of signal preservation, the mean signal intensity per cell as a function of cell surface area and cell volume was evaluated for the different approaches at the same depth of Z = 400 µm for all tissues (Fig. 4A–G). We used the Imaris Surface algorithm to create the 3D reconstructions of microglia according to the maximum signal thresholds in all groups (Fig. 5A–G and Video S1). Because of the differences in tissue expansion across groups, the total cell counts in the selected volume (1024 × 1024 × 400 μm^3^) were corrected and compared. There were no significant differences between total cell counts in the different groups (Fig. 4H). However, the mean intensity of the signals per cell in the FACT group were obviously greater than the other groups (Fig. 4I), and this represents the greatest preservation of fluorophore signal of all the groups during the clearing process.

Because YFP is expressed in the cytoplasm of the microglia in this animal model, the highest volume and surface area of cell bodies represents the highest preservation of fluorophore signals in individual cells. As expected, the selected areas of the FACT-cleared tissues had the maximum amount of cell area and volume in comparison with the other methods (Fig. 5H and I).

Fluorescent bleaching in transgenic samples is an important concern in whole tissue clearing. The presence of free radicals in the tissue clearing process makes the faster processing in FACT approach an important advantage. Aside from reducing tissue clearing time, the optimization of pH and selection of appropriate ingredients for the clearing solvent (PBS) contribute to the ability of FACT to preserve fluorescent signals. In contrast, the use of clearing solutions with a basic pH of 8.5 (CLARITY and SDS 4% without hydrogel) and the inclusion of boric acid contributed to the fading of fluorescent signal in the other approaches.

### Maximal preservation of cytoarchitectural details with FACT

**Figure 6:**
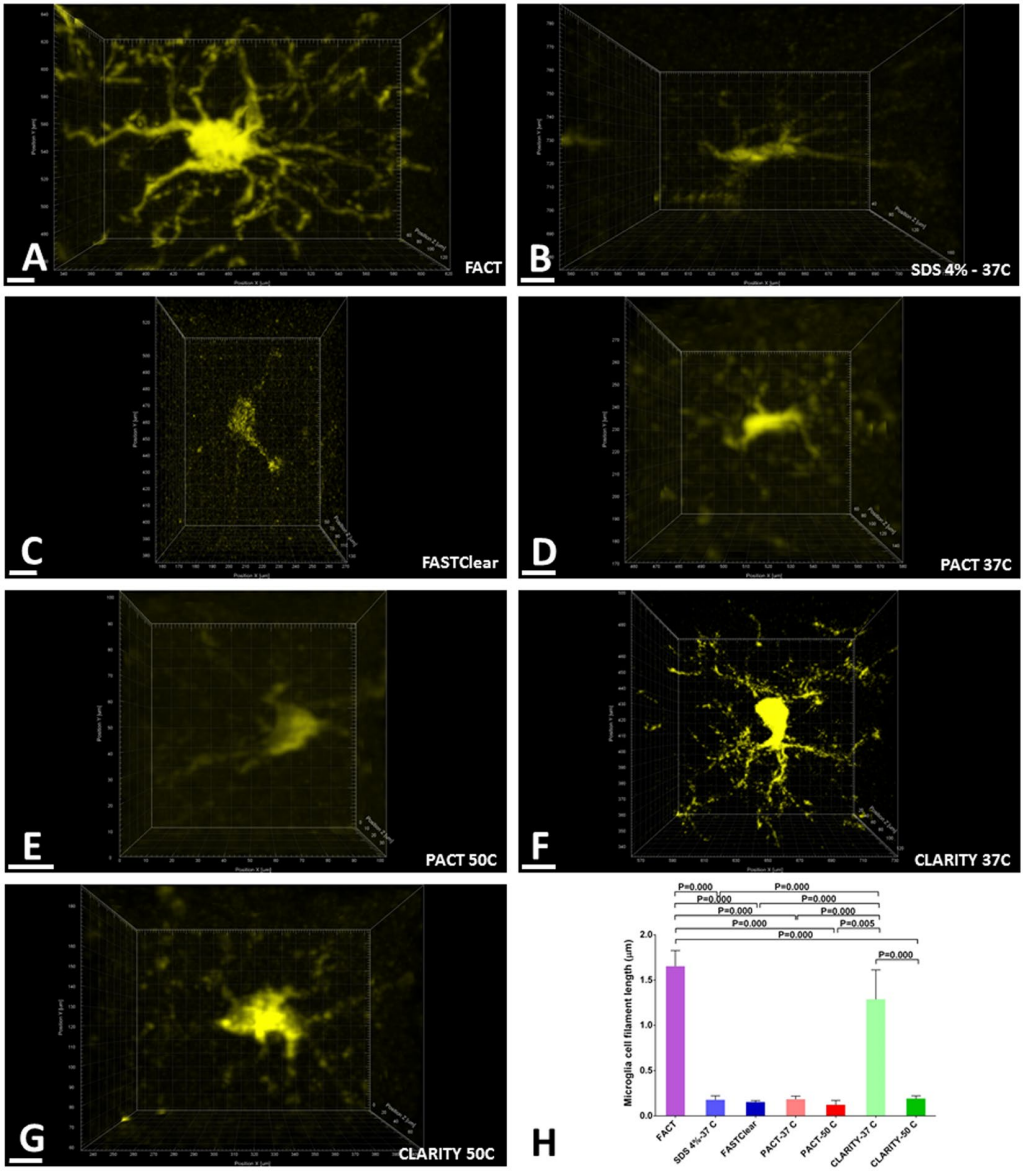
Comparison of the three-dimensional structures of individual microglia. (**A–G**) Selected microglia from the topmost scanned layers (Z = 0–50 μm) of each XYZ acquisition from the different clearing protocols. The finest and most visibly continuous branches are achieved with the FACT protocol. (**H**) Comparisons of microglial branch length among the seven clearing protocols using the semiautomatic Imaris Filament algorithm. The highest signals were detected with the FACT and CLARITY - 37 °C protocols. All confocal images were captured at 25**× **magnification. Scale bars are 20 μm.

**Figure 7:**
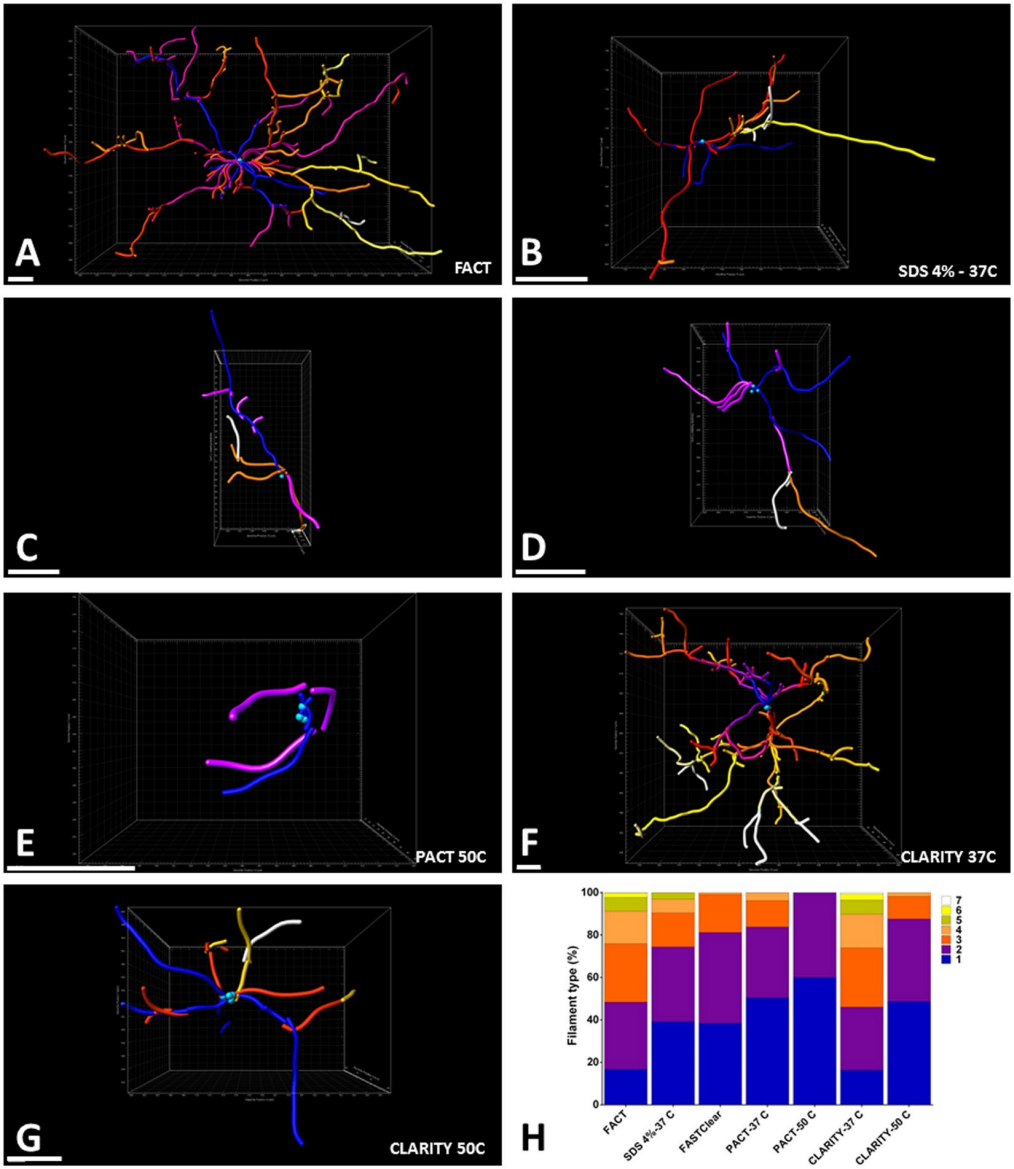
Three dimensional reconstructions of individual microglia to compare the sensitivity of the FACT protocol with the other protocols. (**A**–**G**) Different colors represent different levels of microglial branches reconstructed by the Imaris Filament algorithm and color-sorted by Imaris Vantage from blue (the first level) to white (the last level). Selected microglia from the topmost scanned layers (Z = 0–50 μm) of each XYZ acquisition after the different clearing protocols. (**H**) Comparison of the proportions of 3D reconstructed microglial filament levels for the seven clearing protocols. FACT and CLARITY - 37 °C protocols resulted in the highest degree of branching. Scale bars are 15 μm.

Because microglial cell structure includes a central cell body and several branches and sub-branches that project outward from the soma, we evaluated the ability of the clearing protocol to preserve cell architecture by the detection of continuous signals from branches. For this purpose, complete cells from the surface of the scanned area (Z = 50–100 μm) were individually selected using the 3D crop options of Imaris (Fig. 6A to G), and the Imaris Filament algorithm was used to create the 3D reconstructions of the branches and sub-branches according to the maximum signal thresholds in each cell (Fig. 7A–G). Furthermore, Video S2 illustrates the 3D reconstruction of branches and sub-branches using the Imaris Filament algorithm.

The FACT and CLARITY-37 °C protocols showed the longest branches of all the groups (Fig. 6H). Furthermore, these two approaches demonstrated the maximum variation in branch levels, which is an indication of their ability to preserve the visibility of sub-branches after clearing (Fig. 7H). Due to superior imaging of cytoarchitectural details in deeper tissue with FACT, the user can visualize more detail at both lower (×25) and higher magnifications (×60) compared to the other protocols (Figure S2 and Video S3).

As described above, extreme expansion and irreversible contraction of hydrogel after removing absorbed water from swelled tissue by FocusClear in the hydrogel-based approaches can change cellular microstructures, such as microglia branches. However, the absence of hydrogel from the clearing process in FACT and relying on the cytoskeleton (with its lower water absorbance) for structural support can prevent the deformities caused by polyacrylamide hydrogel expansions.

### Robust antibody labeling of microglia with FACT

**Figure 8:**
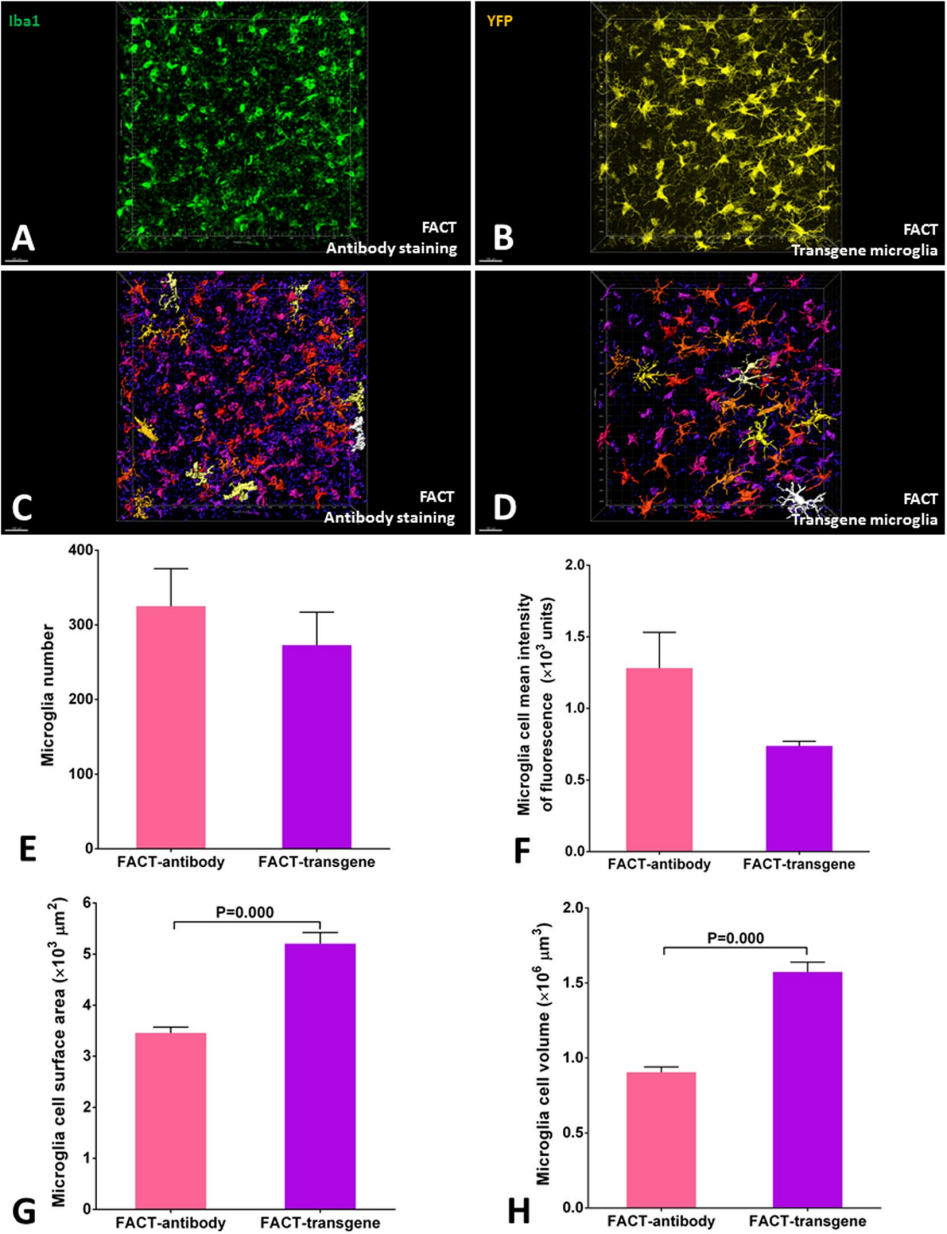
The FACT protocol is compatible with immunohistochemistry and performs well in both antibody-based and transgene-based imaging. (**A** and **B**) Three-dimensional 200-µm-thick volumes of cerebral cortex after antibody staining and YFP-labeled microglia in transgenic mice. (**C** and **D**) Three dimensional reconstructions of microglia surface area and volume by the Imaris Surface algorithm and color sorting of the areas by Imaris Vantage from white (highest area) to purple (lowest area). Higher detection of microglia signal was achieved in the Iba1 immunostaining compared to transgene-YFP-microglia . (**E** and **F**) Comparisons of corrected microglia numbers and corrected mean signal intensities between the two FACT-based detection protocols after semiautomatic counting with the Imaris Surface algorithm. No significant differences were evident (*p* > 0.05). (**G** and **H**) Comparisons of microglial cell surface area and microglial cell volume between the two FACT-based detection protocols after semiautomatic measurement with the Imaris Surface algorithm. Greater signal detection was seen in the antibody-labeledmicroglia compared to the transgene-labeled microglia  (*p* < 0.05). Scale bars are 100 μm.

To test whether the FACT protocol is compatible with antibody staining, we used the Iba1 antibody to stain the brain slices (Fig. 8A) and compared these to transgene-labeled brain slices (Fig. 8B). The microglia populations in the two labeling methods were reconstructed using the Imaris Surface algorithm (Fig. 8C and D). Although the shrinkage of the brain slices after immunostaining was greater than in the transgene-labeled slices, the corrected counting method showed no significant difference in cell number or in mean fluorescent intensity (Fig. 8E and F). However, the greater shrinkage of the slices caused both the cell surface area and the cell volume of FACT-cleared antibody-labeled microglia to be significantly smaller than the FACT-cleared transgene-labeled microglia (Fig. 8G and H).

**Figure 9:**
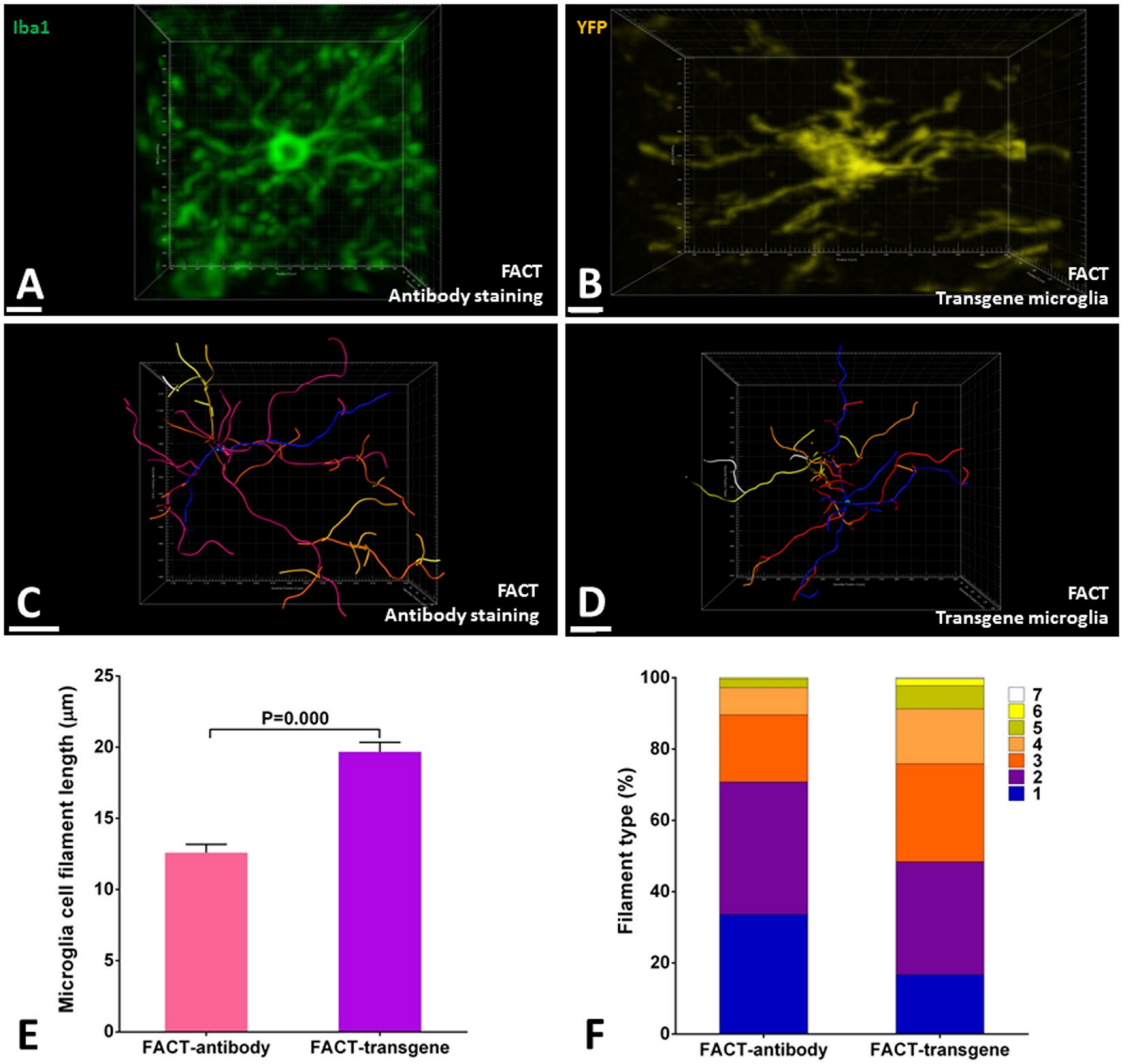
3D structure of individual microglia to compare the sensitivity of the FACT protocol for antibody-stained microglia versus transgene-labeled microglia. (**A** and **B**) Selected microglia from the topmost scanned layers (Z = 0–50 μm) of each XYZ acquisition show fine and continuous branches with both antibody and transgene-YFP signals. (**C** and **D**) Different colors represent different levels of microglia branches reconstructed by the Imaris Filament algorithm and color-sorted by Imaris Vantage from blue (the first level) to white (the last level). Selected microglia from the topmost scanned layers (Z = 0–50 μm) of each XYZ acquisition, representing the maximum types of reconstructed filament levels in the FACT protocol with antibody or transgene-YFP labeling. All confocal images were captured at 25× magnification. (**E**) Comparisons of microglial branch length between the two labeling protocols using the semiautomatic Imaris Filament algorithm. Longer branches were observed in the transgene-YFP microglia compared to antibody labeling due to differences in tissue compaction in the two methods. (**F**) Comparisons of the proportions of 3D reconstructed filament levels. No significant difference was seen between the labeling protocols (*p* > 0.05).Scale bars are 20 μm.

To compare the accuracy of the microglial immunostaining method with transgene-labeled microglia in the FACT-cleared tissue, individual microglia cells from superficial levels of the tissue were cropped (Fig. 9A and B) and analyzed using Imaris Filament after 3D reconstruction (Fig. 9C and D). We found that the mean filament length of antibody-stained microglia was significantly shorter than that of transgene-labeled microglia (Fig. 9E). Because of tissue shrinkage in the immunostaining protocol, and because similar proportions of branches were detected with both labeling protocols (Fig. 9F), the antibody staining exhibited similar architectural details as transgene labeling after the FACT clearing protocol.

Finally, we imaged the striatum of the antibody-labeled brain slices during large image scanning after the FACT (Figure S3A) and CLARITY protocols (Figure S3B) and discovered non-specific hyperchromatic staining of striosomes in the CLARITY protocol but not in the FACT protocol.

In PACT and CLARITY, the hydrogel traps cellular proteins and therefore may also trap antibodies during immunohistochemical procedures. Non-specific binding of primary or secondary antibodies or the trapping of antibodies deep within tissue after washing can increase background staining. Furthermore, non-specific accumulation of antibodies in dense tissues due to the low porosity of the hydrogel may elicit non-specific labeling, such as the staining of striosomes in the CLARITY protocol. In contrast, the absence of the hydrogel in the FACT protocol mitigates these artifacts. Finally, the FACT protocol also increases the depth of antibody penetration and reduces the time spent immunostaining the tissue, as mentioned above.

### Similarity of cell quality and quantity in FACT and thin tissue slicing

Thin sliced brain tissue, as a positive control, was compared with FACT protocol for quantifying the number of fluorescent-labeled microglia and the average intensity of fluorescent signal (Figure S4). Although the background of thin tissue slicing method was higher than FACT but the number of microglia cells and intensity of signals were not different (P > 0.05).

## Discussion

Here we have successfully developed and optimized a new protocol, FACT, for the effective clearing and imaging of both transgene-labeled and immunostained microglia from the telencephalic cortex of the rodent brain. Our systematic comparisons of FACT with seven previously established clearing techniques demonstrate that rapid clearing times as well as preservation of cellular proteins and fine cytoarchitectural details are all achievable with FACT in 1-mm brain slices. The other currently available methods, such as CLARITY^11^, PACT^13^, and SWITCH^19^, are all hydrogel-based, and it has been shown that cellular proteins can be trapped in polyacrylamide hydrogel-embedded tissue by linkage to hydrogel molecules^11^. Furthermore, although Chung *et al*.^11^ and Yang *et al*.^13^ compared their methods with PFA-fixation methods, both groups evaluated CLARITY and PACT with PFA-fixation for the same duration. Thus, our study demonstrates for the first time that brain tissue can be cleared faster in the PFA-based FACT protocol compared to the hydrogel-based CLARITY and PACT protocols, and if the tissues are removed from the clearing solution immediately after optical transparency is achieved or even before complete transparency (as suggested previously for the FASTClear protocol^16^), the protein loss was not different from the CLARITY and PACT protocols. In future studies, investigators may wish to measure the intensity of light transmitted through the slices via brightfield microscopy to further optimize the duration of the clearing steps.

In the present study, we demonstrated for the first time that PFA-fixed transgene-labeled brain tissue can be cleared and imaged with higher tissue quality than either CLARITY or PACT afford. We began our study be contrasting our method to previously published protocols^11, 13, 16^, and observed that our protocol of 8% SDS in PBS (pH 7.5) at 37 °C yielded optimal signal from transgene-labeled microglia. The concentration of SDS in the clearing solution can play an important role in lipid clearance, and it has been shown in hydrogel-embedded tissue that 8% SDS is faster than 4% SDS^13^. However, in the PFA fixation method presented here, SDS does not have the same effect as in hydrogel-based protocols. In addition, the similarity in clearing time between the PACT and CLARITY protocols might be related to the thinner slices (1 mm) used in this study compared to those in the study by Yang *et al*.^13^ (3 mm). Furthermore, we discovered that PBS in the clearing solution has a superior effect on the preservation of endogenous signals than boric acid, and that the pH (7.5 or 7.6) used in the FACT and PACT clearing solutions prevented quenching of fluorophores, while the higher pH (8.5) used in the CLARITY protocol bleached the endogenous fluorophores.

In addition to controlling the pH of the clearing solution to maximize the preservation of endogenous fluorophores, the temperature during the clearing incubation period also plays a critical role in the preservation of fluorescent signal. The high temperature tested in this study (50 °C) increased the rate of lipid clearing from the tissue, but it also led to increased protein loss, increased tissue expansion, and decreased imaging depth for the endogenous florescent signals. Thus, we conclude that 37 °C is the optimum temperature for all protocols tested here.

Another concern we attempted to address was to establish a method that not only reduces the clearing time of the brain tissue, but does so without damaging the fine structures of microglial branches and sub-branches. Reducing the tissue size change ratio for transgene-labeled microglia during clearing, reducing the sample thickness in the immunostaining protocol, and removing hydrogel from the fixation protocol all served to reduce cellular deformations during the imaging step and provided a clear picture of cellular architecture with both labeling methods. Considering that microglia are smaller than neurons in the CNS, the FACT protocol should theoretically render it possible to image both populations of cells, their fine processes, and their structural interactions. Although it has been reported that removing ETC from the clearing protocol avoids the tissue degradation seen with the passive CLARITY^12^ and PACT protocols^13^, our findings demonstrate that hydrogel deformation is another factor that can change the fine structures of cells, such as microglial branches.

In terms of optimizing the immunostaining of FACT-cleared brain slices, we speculate that removing polyacrylamide hydrogel increased the speed, amount, and depth of antibody penetration and therefore provided a clear image comparable to endogenous fluorescent labels. Consistent with our findings in the modified CLARITY protocol, reducing the polyacrylamide hydrogel concentration from 4% to 1% increased the penetration of antibodies^20^. Furthermore, trapping of either the primary or secondary antibodies in the hydrogel networks or crosslinking of these antibodies with the hydrogel caused unexpected non-specific staining and increased background during the imaging, which significantly reduced image quality. Consistent with our study, it has been reported that the preservation of antigenicity is not optimal in the CLARITY protocol^21^, but the reason for this was not discussed. Here we show that the removal of hydrogel in the FACT protocol circumvents this limitation of previous methods.

To make a method usable for the greatest number of researchers, consideration must be given to various criteria, such as the price of materials, the safety of the reagents for users and the environment, the simplicity of the protocols, the amount of labor required, and the equipment that is needed, in addition to the quality of the histology. FACT, compared with the other methods evaluated in this study, performs well in all of these areas. All of the approaches reported here employed light microscopy, which is limited to submicron resolution due to the working distance of the optical lens in both confocal and light sheet microscopes. Therefore, imaging of normal-sized microglia with a depth range of 300 μm to 700 μm should be achievable at micron resolution using available microscopes, and FACT was the only protocol tested here that still exhibited strong signal detection at these depths.

Together with its simplicity, cost-effectiveness, and high speed of clearing, our data suggest that the FACT protocol will prove invaluable for future studies of the fine cytoarchitectural details of brain cells such as microglia. Such studies are expected to improve our understanding of physiological and pathological conditions and their effects on protein expression in structures ranging in size from fine cellular sub-branches to global neuronal networks spanning across the CNS.

## Methods

### Animals

C57BL/6 N mice (Laboratory Animal LLC, Shanghai, China) and B6.129P2(Cg)-Cx3cr1^tm2.1(cre/ER,-EYFP)Litt^/WganJ mice (The Jackson Laboratory) were used for breeding and for all experiments. Handling of the animals and all experimental methods were performed according to the Animal Research Ethic guidelines of Fudan University, which conforms to international guidelines. All experiments were approved by the Institute of Brain Science research committee of Fudan University (Shanghai, China).

Adult wild type and microglia-yellow fluorescent protein (YFP) transgenic mice (8 weeks old) were anesthetized with intraperitoneal injections of pentobarbital (0.35 mg/kg). The mice were perfused transcardially with either hydrogel monomer solution (for the passive CLARITY and PACT protocols) or paraformaldehyde (PFA) solution (for the FACT and FASTClear protocols). The mouse brains were removed, and 1 mm coronal slices were cleared with one of the studied techniques (Fig. 1). To prevent fluorescent photobleaching after brain sampling and during the clearing procedure, the samples were processed in tubes that were covered with aluminum foil, and all procedures were performed in a dimly lit room.

### The FACT protocol

Mice were transcardially perfused with 40 mL ice-cold phosphate-buffered saline (PBS) solution (1 M, pH 7.6) followed by 20 mL of 4% (wt/vol) PFA in 1 M PBS. After collecting 1 mm coronal sections with a mouse brain mold, the brain slices were post-fixed in the same fixative solution at 4 °C for 3 days. The slices were randomly allocated into four groups (Fig. 1). The slices were cleared with either 8% (wt/vol) SDS in 0.1 M PBS (pH 7.5) or 4% (wt/vol) SDS in sodium borate buffer (200 mM; pH 8.5), either at 37 °C or 50 °C with gentle rotational shaking. The FACT subgroup was treated with 8% SDS clearing solution at 37 °C, and the FASTClear subgroup was treated with 4% SDS clearing solution at 50 °C. The solutions were refreshed daily until visual confirmation of complete tissue transparency by viewing black grid lines on a white sheet of paper through the tissue itself.

### PACT protocol

Transcardial perfusion was carried out with 40 mL ice-cold PBS solution (1 M, pH 7.6) followed by 20 mL of a mixture of 4% (wt/vol) PFA, 4% (wt/vol) acrylamide, and 0.25% (wt/vol) VA-044 initiator in Millipore double-distilled water (Fig. 1). The dissected brains were incubated in the same hydrogel monomer solution at 4 °C for 3 days. The samples were then degassed by filling the tubes with fresh hydrogel monomer solution in a shaking incubator at 37 °C to initiate polymerization. The embedded brains were removed from the hydrogel after the gel solidified (maximum 3 h), and after removing excess hydrogel on the brain surface with tissue paper, brains were cut into 1 mm coronal sections. The samples were incubated with clearing solution containing 8% SDS in 0.1 M PBS (pH 7.5) at either 37 °C or 50 °C with gentle rotational shaking. The PACT solution was refreshed daily for 3 days and then was changed weekly until complete transparency was achieved. The transparency of the tissue was checked daily.

### Passive CLARITY protocol

Similar sampling procedures were carried out, and the same hydrogel monomer solutions as in the PACT method were used up until the clearing step (Fig. 1). The samples were divided into two groups, and lipid wash-out was achieved by passive clearing in a solution of 200 mM sodium borate buffer (pH 8.5) containing 4% (wt/vol) SDS at either 37 °C or 50 °C with gentle rotational shaking. The passive CLARITY solution was refreshed daily for 3 days, and then it was changed weekly until complete transparency was achieved. The transparency of the tissue was checked on a daily basis.

### Antibody staining of FACT-cleared tissue

After clearing of wild-type brain slices with the FACT protocol, the SDS was removed by washing in PBS with 0.1% Triton X-100 (PBST) for 12 h. PBST solution was replaced every 6 h. The tissues were permeabilized and blocked overnight at 37 °C with 0.6 M glycine, 0.2% Triton X-100, 6% donkey serum, and 20% dimethyl sulfoxide (DMSO) dissolved in PBS. The tissues were washed two times in PBST for 1 h at 37 °C. The washed brain slices were incubated with primary antibody (Iba1, Abcam) diluted 1:50 in 0.2% Tween-20, 5% DMSO, 3% donkey serum, and 0.01% sodium azide in PBS for a minimum of 2 days at 37 °C. The samples were washed again three times in PBST for 1 h and incubated overnight at 37 °C. Sections were then incubated with secondary antibodies (Alexa594) diluted 1:200 in 0.2% Tween-20, 5% DMSO, 3% donkey serum, and 0.01% sodium azide in PBS for a minimum of 2 days at 37 °C. All procedures were conducted with shaking at 37 °C. The samples were washed again three times in PBST for 1 h each, and the tissues were then incubated overnight at 37 °C or transferred to a refrigerator (4 °C) where they remained for 7 days in aluminum foil-covered tubes containing PBST and 0.01% sodium azide.

### Antibody staining of passive CLARITY-cleared tissue

After clearing wild-type mouse brain slices with the passive CLARITY protocol, the residual SDS was removed from the brain slices by slowly shaking in PBST for 24 h. Samples were then incubated with primary antibody (Iba1, Abcam) diluted 1:50 in PBST for 2 days. The samples were washed in PBST buffer for 1 day, followed by exposure to secondary antibodies (Alexa594) diluted 1:200 in PBST for 2 days. Before mounting and imaging, samples were washed in PBST for at least 1 day. All procedures were conducted with shaking at 37 °C. For the 7 days between immunostaining and imaging, the labeled slices were transferred to a refrigerator (4 °C) where they were kept in aluminum foil-covered tubes containing PBST and 0.01% sodium azide.

### Refractive index homogenization

After clearing, the brain slices from each group were either incubated at 4 °C (for a maximum of 3 weeks for transgene-labeled slices or 1 week for antibody-labeled slices) until imaging, or the cleared tissues were transferred directly into FocusClear (CelExplorer Labs) for 1 h prior to imaging.

### Comparisons of FACT and thin tissue slicing

To prepare thin sliced brain samples, mice were transcardially perfused with 40 ml PBS solution (1 M, pH 7.6) followed by 20 ml of 4% (wt/vol) PFA in PBS. Brains were harvested and cryoprotected in 30% sucrose in PBS for two days. Frozen serial coronal brain sections (30-μm thick) were prepared on a cryostat (Microm HM459, Thermo Scientific). Brain Sections were mounted and cover slipped. Images were captured with a Nikon A1R+ upright confocal microscope. Firstly, the laser was focused onto the specimen by with an oil immersion 25× objective lens, the objective lens was placed on the cortex area and three fields without Z depth were imaged. Image analysis was done using Photoshop CS6 (Adobe® Photoshop®). Average intensity of microglia signals and number of cells were measured^22^.

### Confocal microscopy

The brain slices were embedded in a chamber formed by a 1-mm thick and flattened horse-shoe-like piece of putty acting as a wall on a glass slide. The chamber was filled with FocusClear, and the upper part of the chamber was gently sealed using a Wellco dish (Pelco (Ted Pella), cat. no. 14032E120) with the glass surface facing down and thus preventing the formation of small bubbles on the surface of the brain slice. We used a Nikon A1R^+^ upright confocal microscope to obtain all of the confocal images presented here. After fixing the embedded apparatus on the microscope stage, the laser was focused onto the specimen with a water immersion 25× objective lens (1.1-NA, 2 mm-WD, Nikon, USA). After defining the edges of the tissue slice and capturing a large-scale image, the objective lens was placed on the cortex area and three fields (XY = 1024 × 1024 μm^2^) with whole tissue depth (Z = maximum visible signals down to 1000 μm) were scanned (speed = 0.5, step distance = 1 μm). Prior to Z-scanning, the laser power, light gain, and offset of the upper and lower visible surfaces of the slice were defined for the highest acquisition of excitation and emission of microglial YFP signals using the *intensity correction* option of the Nikon NIS software. After obtaining the images of the brain cortex, the TIFF image sequences were transferred to Bitplane Imaris software (version 7.4.2) for 3D reconstruction and image analysis.

### 3D reconstruction and image analysis

The 3D reconstruction and tracing of microglial morphology was performed using Imaris software and its algorithms, including Surface and Filament, and automatic or semiautomatic counting. Because of the large amount of data, a workstation server was used for the data analysis with the following configuration: Dell server board T7910, two Intel E5-2687WV4 CPUs, four ~32 GB DDR4 ECC RAM, a ~4 TB hard disk (Dell SAS 7.2 K), and an NVidia Quadro 5000 graphics card.

The 3D reconstruction of the cell surface based on the signal thresholds of microglia cells was performed in two steps in Imaris Surface. In the first step, an index was defined and measured for all of the Z-depth images in order to show the similarity of the number of microglia cells in different parts of the cortex and to remove the effect of tissue expansion when counting the numbers of cells per unit volume. For this purpose, we counted the numbers of cells in three virtual squares using the *region of interest* option of the Imaris Surface software (XYZ = 200 × 200 × 100 μm^3^ from the surface layer). The FACT-cleared cortex, which had the highest numbers of cells, was selected as the reference, and the averages of the cell counts in all groups were divided by the average of the reference counts. In the next step, using the *entire image* option of Imaris Surface, the cells in each of the Z-depth images were reconstructed (surface area detail = 0.2 μm and manual adjustment of threshold to cover the whole visible microglia volume). After counting the cells, the total cell numbers were corrected by multiplying the calculated number index of the first step in each sample to measure the total cell number in each imaged volume. Furthermore, the data for the cell surface area and the mean signal intensity in the detected cells were extracted from the Imaris Surface analysis of the entire images and evaluated as indices of cell signal quality with the different clearing protocols. Finally, the cells were categorized and imaged in Imaris Vantage according to the cell surface area.

Imaris Filament was used to determine the accuracy of the methods in detecting the fine branches of microglial cells. For this purpose, three complete cells that were imaged at the 100 μm depth of the whole image were selected using the 3D crop option of Imaris Filament. We then used the semiautomatic settings to reconstruct the cell centers and filaments of the selected cells, and we analyzed the filament levels and lengths as indices of the visibility of the branches and sub-branches of the individual cells in each method. Using Imaris Vantage, the 3D reconstructed images were classified according to the filament levels.

To compare antibody-stained and transgene-labeled microglia in the FACT protocol, the same image analysis methods were performed with the Imaris Surface and Filament algorithms. Z-depths of 200 μm were evaluated in both methods.

### Sliced tissue expansion and weight gain measurement

The 1-mm coronal slices from one half of the brain were cleared with the techniques described above. Slices (n = 3) were weighed and imaged with a conventional camera before, during, and after clearing and after refractive index homogenization in FocusClear. The slices were outlined and their sizes were calculated using ImageJ software. The tissue expansion and weight gain were determined by calculating the change in size and weight of slices after clearing and after immersion in FocusClear for 1 h.

### Protein loss measurement

The percentage of protein loss for each sample (n = 3) was obtained by measuring the amount of total protein in the clearing solutions collected daily from all groups until clearing. Protein content was assessed with the xMark™ Microplate Absorbance Spectrophotometer blanked with the respective solutions and normalized to the weight of the slices before clearing.

### Statistical analysis

The data are presented as bar charts (means ± standard error of means), box plots, or proportion bar plots (GraphPad Prism version 6.00 for Windows, GraphPad Software, La Jolla, CA, USA). Comparisons between the data were performed using one-way ANOVA with Tukey’s *post hoc* test (for comparing different clearing approaches), independent sample *t*-test (for comparing antibody and transgenic labeling with the FACT protocol and comparing FACT and thin tissue slicing), or chi-square test (for comparing the proportions of microglia branches) in IBM SPSS Statistics for Windows (Version 22.0, 2013; IBM Corp., Armonk, NY). A value of *p* ≤ 0.05 was set as the limit for statistical significance.

## Electronic supplementary material

Supplementary materials

Video S1

Video S2

Video S3
