# Supplementary material for: Fast free-of-acrylamide clearing tissue (FACT)—an optimized new protocol for rapid, high-resolution imaging of three-dimensional brain tissue
Source: Sci Rep. 2017 Aug 29;7:9895. doi: 10.1038/s41598-017-10204-5 (PMC5575133; doi:10.1038/s41598-017-10204-5)
Supplement: Supplementary file 1 — Supplementary materials [file 41598_2017_10204_MOESM1_ESM.pdf]

Fast free-of-acrylamide clearing tissue (FACT)—an optimized new protocol for rapid, high-resolution imaging of three-dimensional brain tissue

Na Xu, Amin Tamadon, Yaan Liu, Tong Ma, Rehana K. Leak, Jun Chen, Yanqin Gao, Yi Feng

Supplementary materials

A) Supplementary Figures

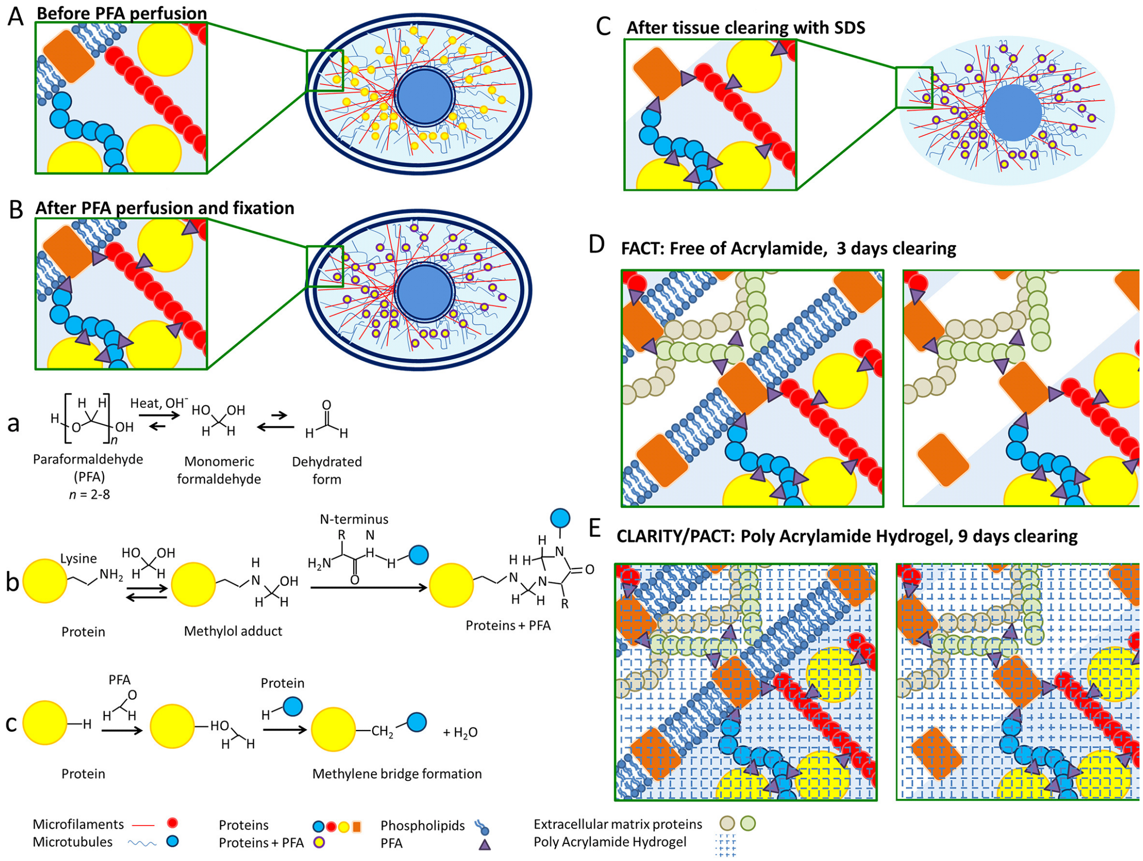

A Before PFA perfusion
B After PFA perfusion and fixation
C After tissue clearing with SDS
D FACT: Free of Acrylamide, 3 days clearing
E CLARITY/PACT: Poly Acrylamide Hydrogel, 9 days clearing
a
Heat, OH-
Paraformaldehyde (PFA) n = 2-8
Monomeric formaldehyde
Dehydrated form
b
Lysine
N-terminus
Protein
Methylol adduct
Proteins + PFA
c
PFA
Protein
Methylene bridge formation
Microfilaments
Microtubules
Proteins
Proteins + PFA
Phospholipids
PFA
Extracellular matrix proteins
Poly Acrylamide Hydrogel

Figure S1. Mechanisms underlying the efficacy of the Fast Free-of-Acrylamide Clearing Tissue (FACT) protocol. (**A-B**) During the paraformaldehyde (PFA)-mediated tissue fixation in the FACT protocol, membrane and intracellular cytoplasmic proteins (including the transgenic fluorescent proteins) make chemical bonds with the cytoskeleton, including microfilaments and microtubules, and/or with the extracellular matrix, including proteoglycans. These bonds help construct a massive 3D matrix that lends structural support and tensile strength to the tissue during processing. (**C**) After removing the cell walls with 8% SDS in pH 7.5 (optimum pH for preserving normal protein structures), the tissue scaffold is chemically bonded by PFA. (**D-E**) Compared to CLARITY and PACT, absence of the hydrogel in the FACT protocol facilitates lipid clearance during incubation with SDS. The polyacrylamide hydrogel in the CLARITY or PACT protocols may trap proteins (including antibodies) chemically or physically and creates an obstacle for fast removal of lipid.

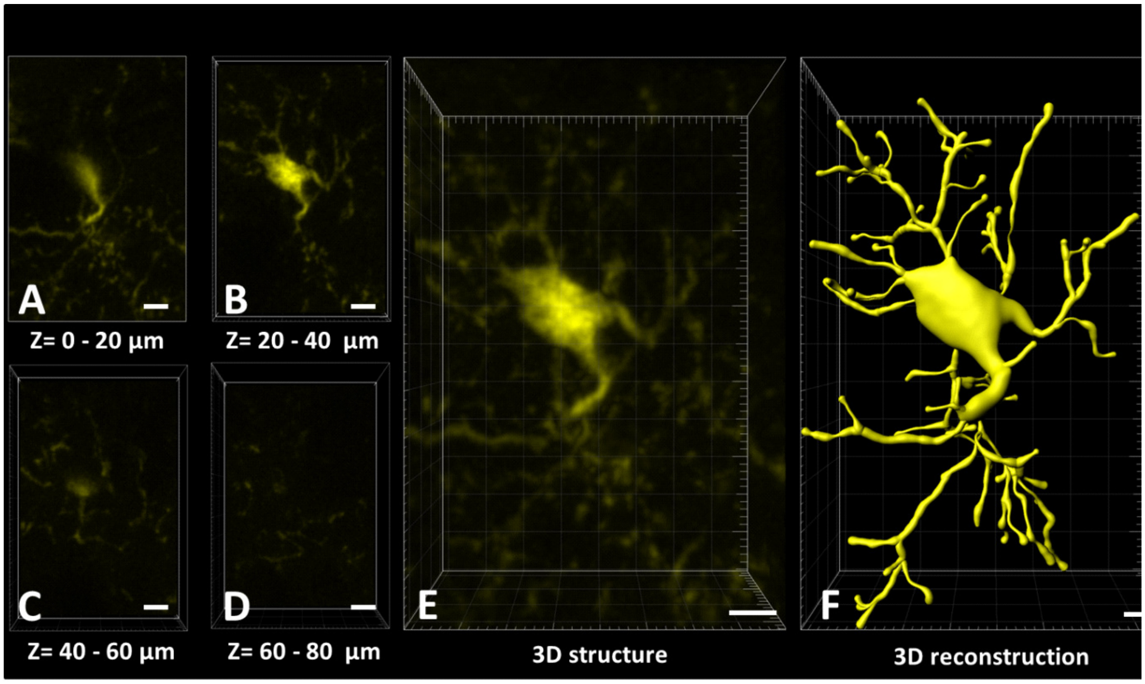

Figure S2. **3D reconstruction of a YFP-expressing microglial cell in the cerebral cortex of transgenic mice.** Brain slices were cleared with the Fast Free-of-Acrylamide Clearing Tissue (FACT) protocol, imaged at 60× magnification, and processed with the

Imaris Surface algorithm and Filament algorithm. (**A-D**) 20 μm-thick sections of a microglial cell at 80 μm depth (z). (**E**) 3D structure of microglia. (**F**) 3D reconstruction of the same microglia with Imaris Surface algorithm. Scale bars are 20 μm.

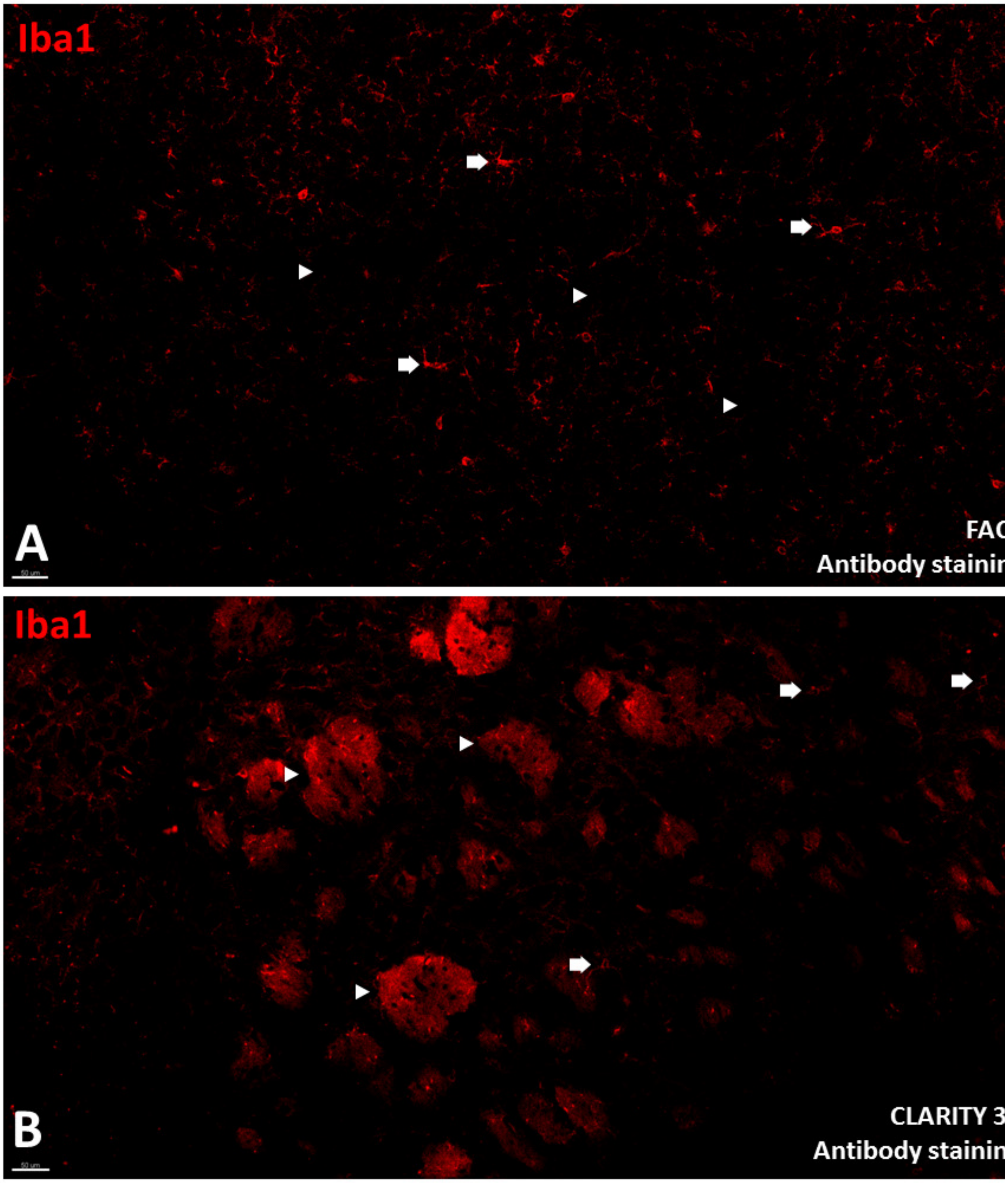
Iba1
A
FAC
Antibody stainir
Iba1
B
CLARITY 3
Antibody stainin

Figure S3. Antibody staining of microglia (arrows) in the striatum with the Fast Free-of-Acrylamide Clearing Tissue (FACT) and CLARITY 37° C protocols. (A) In the FACT protocol there was no non-specific hyperchromatic staining of striosomes in the caudoputamen (arrowheads). (B) In the CLARITY 37° C protocol, non-specific hyperchromatic staining of striosomes was evident (arrowheads). Confocal large-scan imaging at 25× magnification. Scale bars are 50 μm.

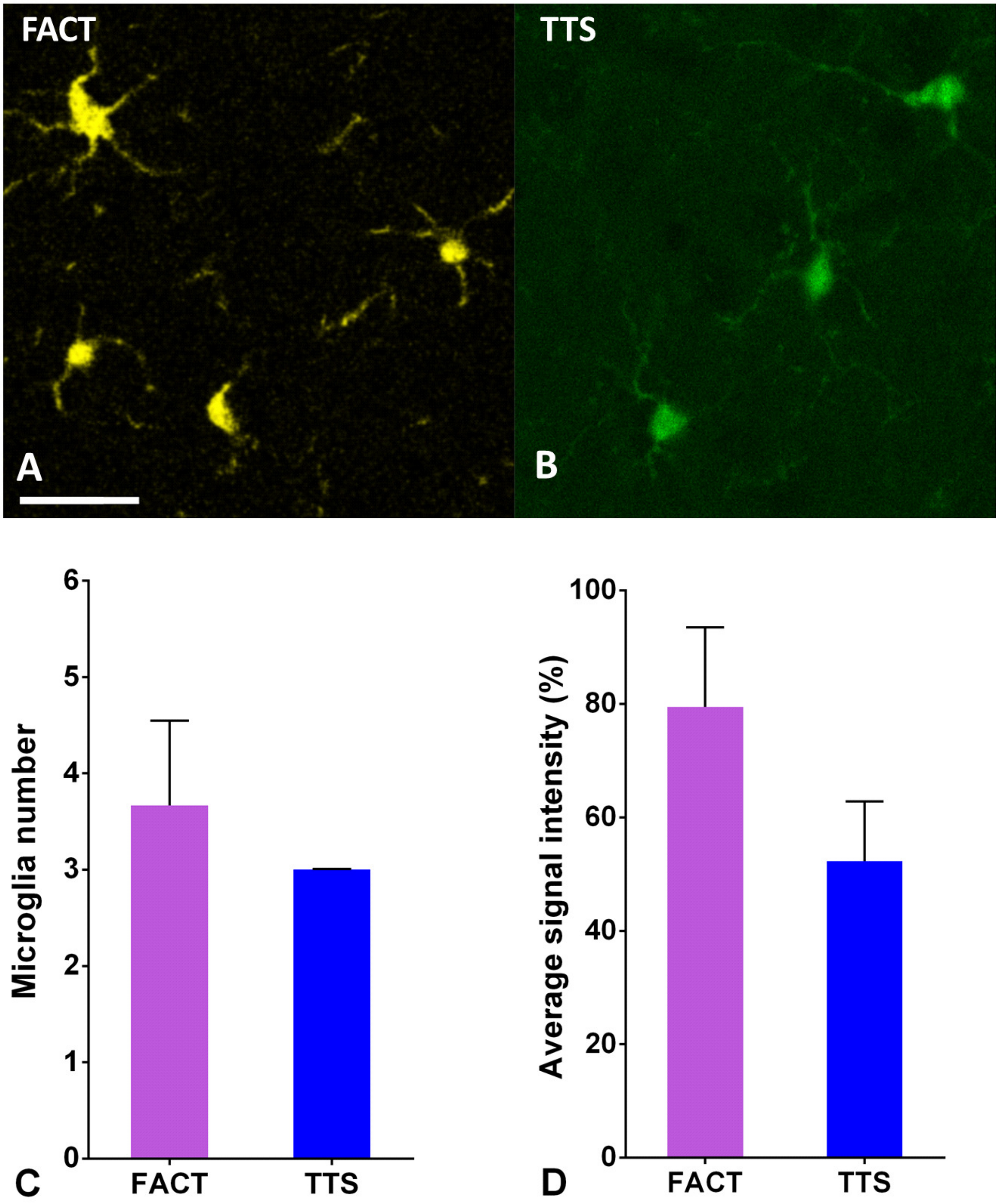
FACT
TTS
A
B
6
5
4
3
2
1
0
Microglia number
C
FACT
TTS
100
80
60
40
20
0
Average signal intensity (%)
D
FACT
TTS

Figure S4. Comparison of Fast Free-of-Acrylamide Clearing Tissue (FACT) and thin tissue slicing (TTS). (**A**) The FACT protocol. (**B**) The TTS protocol. (**C**) Mean and SE of number of microglia cells in a same field of imaging. (**D**) Mean and SE of average of fluorescent intensity (%) in the imaged microglia. Confocal large-scan imaging at 25× magnification. Scale bars are 100 μm.

B) Supplementary Videos

Video S1. **High-resolution imaging of microglia expressing YFP in the cerebral cortex of transgenic mice**. Sections were rapidly cleared with the Fast Free-of-Acrylamide Clearing Tissue (FACT) protocol and 3D surface reconstruction and analysis were performed with Imaris Surface algorithm and Imaris Vantage.

Video S2. **3D reconstruction of YFP-expressing microglial cells in the cerebral cortex of transgenic mice.** Sections were cleared with Fast Free-of-Acrylamide Clearing Tissue (FACT) protocol and processed with the Imaris Filament algorithm.

Video S3. **3D reconstruction of an YFP-expressing microglial cell in the cerebral cortex of transgenic mice**. Brain slices were cleared with the Fast Free-of-Acrylamide Clearing Tissue (FACT) protocol, imaged at 60× magnification, and processed with the Imaris Surface algorithm and Filament algorithm.
